# Distal Ureteric Stone Presenting Solely As Testicular Pain: Diagnostic Lessons From a Primary-Care Setting

**DOI:** 10.7759/cureus.96289

**Published:** 2025-11-07

**Authors:** Ayyaz Mulla, Fayaz Mulla, Tarun Odur, Shabbeer Kaggal, Rohil Kumar

**Affiliations:** 1 General Practice, R.R. Sugar, Fever and Dental Clinic, Penukonda, IND; 2 Internal Medicine, People's Education Society (PES) Institute of Medical Sciences and Research (PESIMSR), Kuppam, IND; 3 General Medicine, Sagar Hospitals, Bangalore, IND; 4 General Practice, SL AP Pvt Ltd, Penukonda, IND

**Keywords:** conservative management, distal ureter, hydroureteronephrosis, primary care, tamsulosin, testicular pain, ultrasound, ureteric stone

## Abstract

Ureteric stones are a common cause of acute flank pain, but their presentation can vary widely depending on the stone’s size and location. Pain may radiate to the groin or testis because of shared sensory innervation between the ureter and the genitofemoral and ilioinguinal nerves. However, isolated testicular pain without urinary or flank symptoms is a rare presentation and can easily lead to misdiagnosis in primary-care settings.

We report the case of a 23-year-old man who came to a small primary-care clinic with sudden right-sided testicular pain. Examination and scrotal ultrasound were both normal, ruling out torsion and infection. Because the pain persisted, a quick abdominal ultrasound was arranged, which showed mild right-sided hydroureteronephrosis and about 5 mm echogenic focus near the vesico-ureteric junction, suggesting a distal ureteric stone. The patient was managed conservatively with oral fluids, analgesics, and tamsulosin 0.4 mg once daily. Within three days, he passed the stone and became symptom-free. A repeat scan a week later confirmed complete resolution.

This case highlights the importance of considering distal ureteric stones as a differential diagnosis for unexplained testicular pain, especially in primary-care and resource-limited environments where early imaging can prevent misdiagnosis and unnecessary interventions.

## Introduction

Ureteric stones are a common problem in adults, affecting about 10%-12% of people worldwide [1,2]. They are seen more often in men between 20 and 50 years of age and may cause flank or lower abdominal pain, hematuria, and urinary symptoms depending on the stone's size and position [3,4]. When the stone is located in the lower ureter, the pain may sometimes be felt in the groin or testis because of the shared nerve supply [5]. Rarely, a small distal stone may present only as scrotal or testicular pain, which can be confusing for doctors in a primary-care clinic [6,7].

The main clinical features of distal ureteric stones include sudden sharp pain, nausea, vomiting, and blood in the urine. However, isolated scrotal pain may mimic testicular torsion, epididymitis, or varicocele [8,9]. Careful examination, urine testing, and simple imaging are important to rule out these urgent conditions [10,11].

Ultrasound is a safe, quick, and easily available first-line investigation, especially where CT scans are not accessible or are too expensive [12,13]. Although CT remains the gold standard for small stones, ultrasound can still identify hydronephrosis, stone location, and later confirm clearance [14,15].

Most stones smaller than 5 mm pass on their own with hydration, analgesics, and alpha-blockers such as tamsulosin [16,17]. Larger stones may need endoscopic or laser procedures [18,19]. Delay in diagnosis can lead to infection, obstruction, and loss of kidney function [20].

This report describes a rare presentation of a distal ureteric stone as isolated testicular pain. It reminds clinicians to consider urinary causes in patients with unexplained scrotal pain and shows how early ultrasound use in a primary-care setup can help avoid unnecessary anxiety, referrals, and hospital visits, supporting efficient and patient-centred care in community settings.

## Case presentation

A 23-year-old male patient visited a general-practice clinic in southern India complaining of sharp right-sided testicular pain lasting six hours. The pain radiated toward the groin and was not linked with fever, vomiting, or injury. There was no dysuria, visible blood in the urine, or previous stone disease.

On examination, both testes were normal in size and consistency, with no swelling, redness, or scrotal erythema. There was no tenderness over the epididymis or spermatic cord, and the cremasteric reflex was intact. Mild right lower-quadrant discomfort was felt on abdominal palpation without guarding.

**Figure 1:**
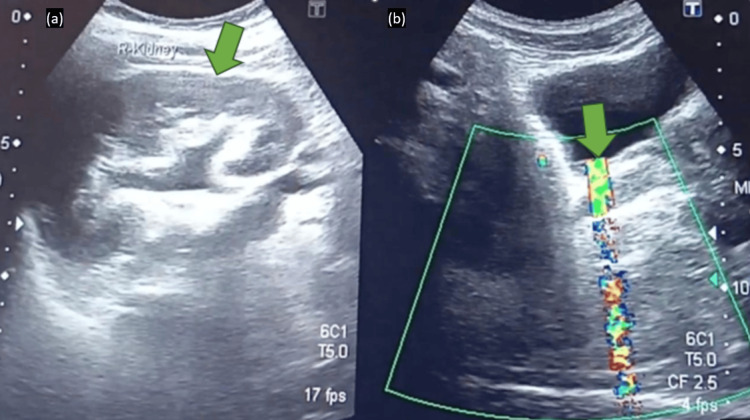
Ultrasound Showing Mild Right-Sided Hydroureteronephrosis and Distal Ureteric Echogenic Focus (a) Ultrasound image showing mild right-sided hydroureteronephrosis (green arrow). (b) Ultrasound image demonstrating a distal ureteric echogenic focus at the vesico-ureteric junction (green arrow) with posterior acoustic shadowing and color-Doppler twinkling artefact, consistent with an impacted calculus.

**Figure 2:**
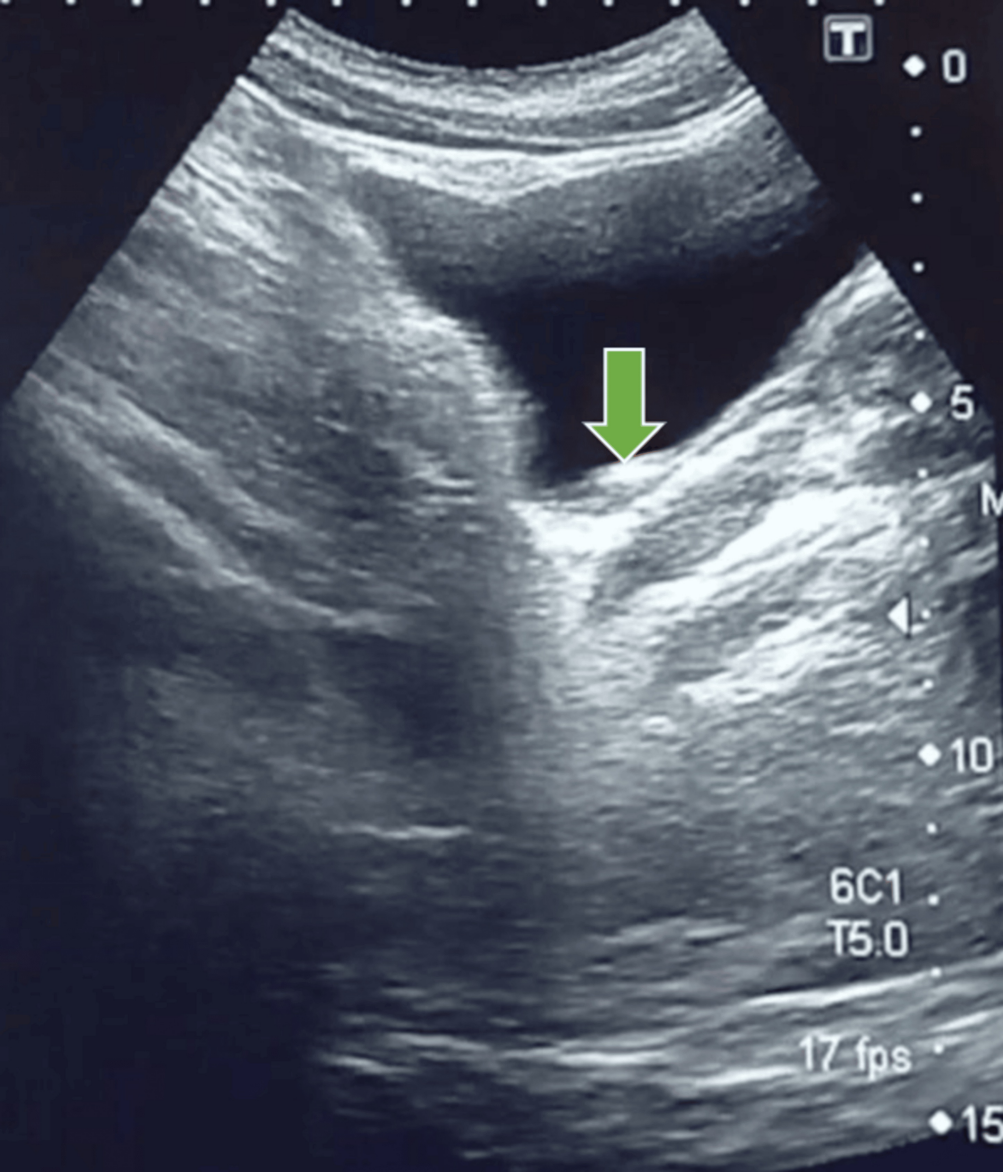
Ultrasound Demonstrating Distal Ureteric Calculus with Mild Upstream Dilatation Ultrasound of the pelvis demonstrating an echogenic focus with posterior acoustic shadowing at the right vesico-ureteric junction (green arrow), consistent with about 5 mm distal ureteric calculus causing mild upstream dilatation.

Urine dipstick showed microscopic haematuria. Serum urea and creatinine were normal. Scrotal ultrasound showed both testes of normal size and echotexture with preserved intratesticular blood flow and no evidence of torsion, epididymal thickening, or hydrocele. A renal and bladder ultrasound done at a nearby diagnostic centre by a qualified radiologist showed mild right-sided hydroureteronephrosis and about 5 mm echogenic focus at the vesico-ureteric junction with posterior acoustic shadowing and colour-Doppler twinkling artefact, findings typical of a distal ureteric calculus (Figures 1, 2) [6].

**Figure 3:**
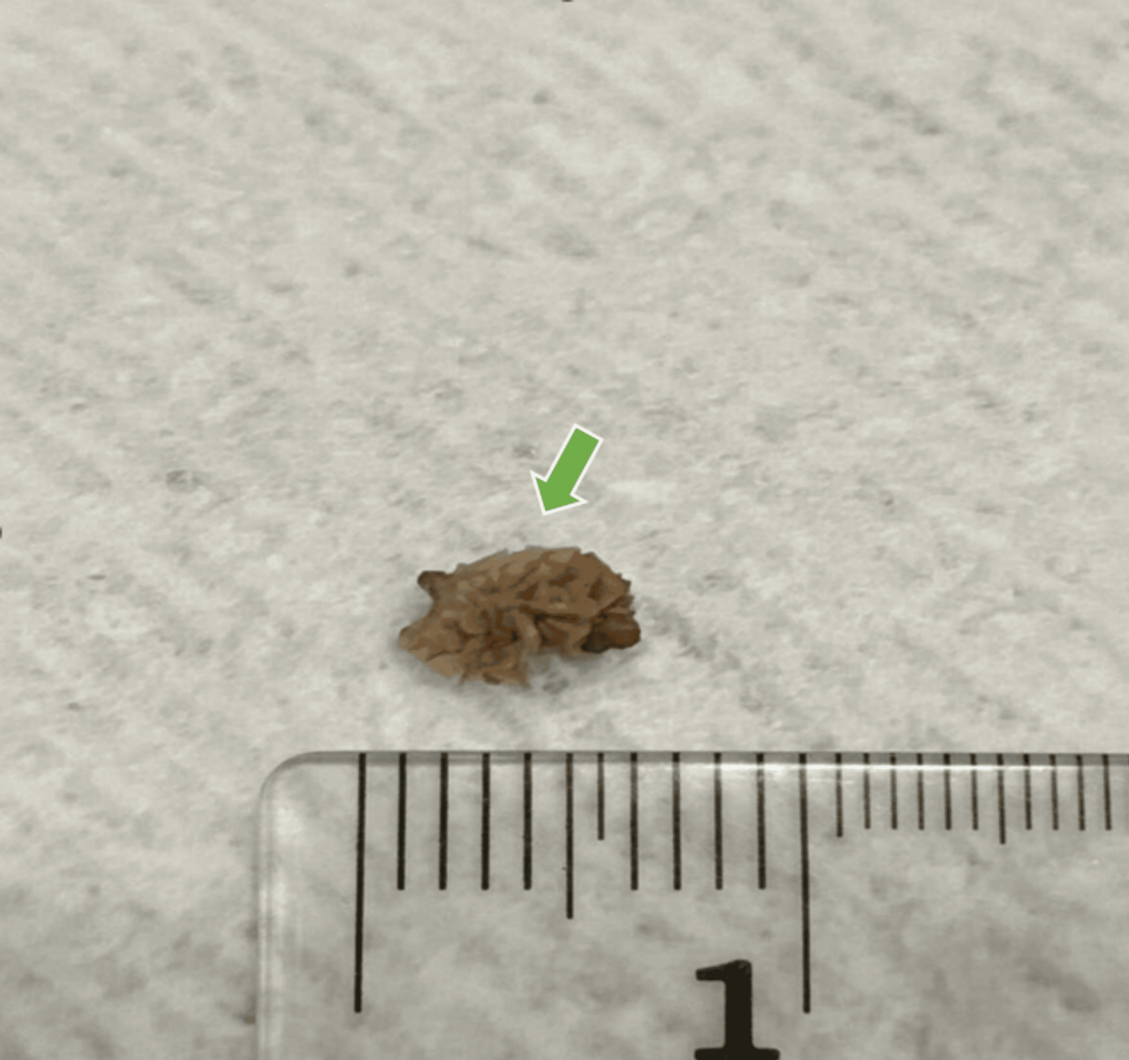
Photograph of the Spontaneously Passed Ureteric Stone Gross image of the spontaneously passed ureteric calculus (green arrow) measuring approximately 5 mm in maximal diameter. The stone was retrieved by the patient during urination following medical expulsive therapy and measured against a millimetre scale for reference.

He was advised good oral hydration and started on tamsulosin 0.4 mg daily with simple analgesics [7]. Within three days, he passed a small calculus with complete pain relief (Figure 3).

**Figure 4:**
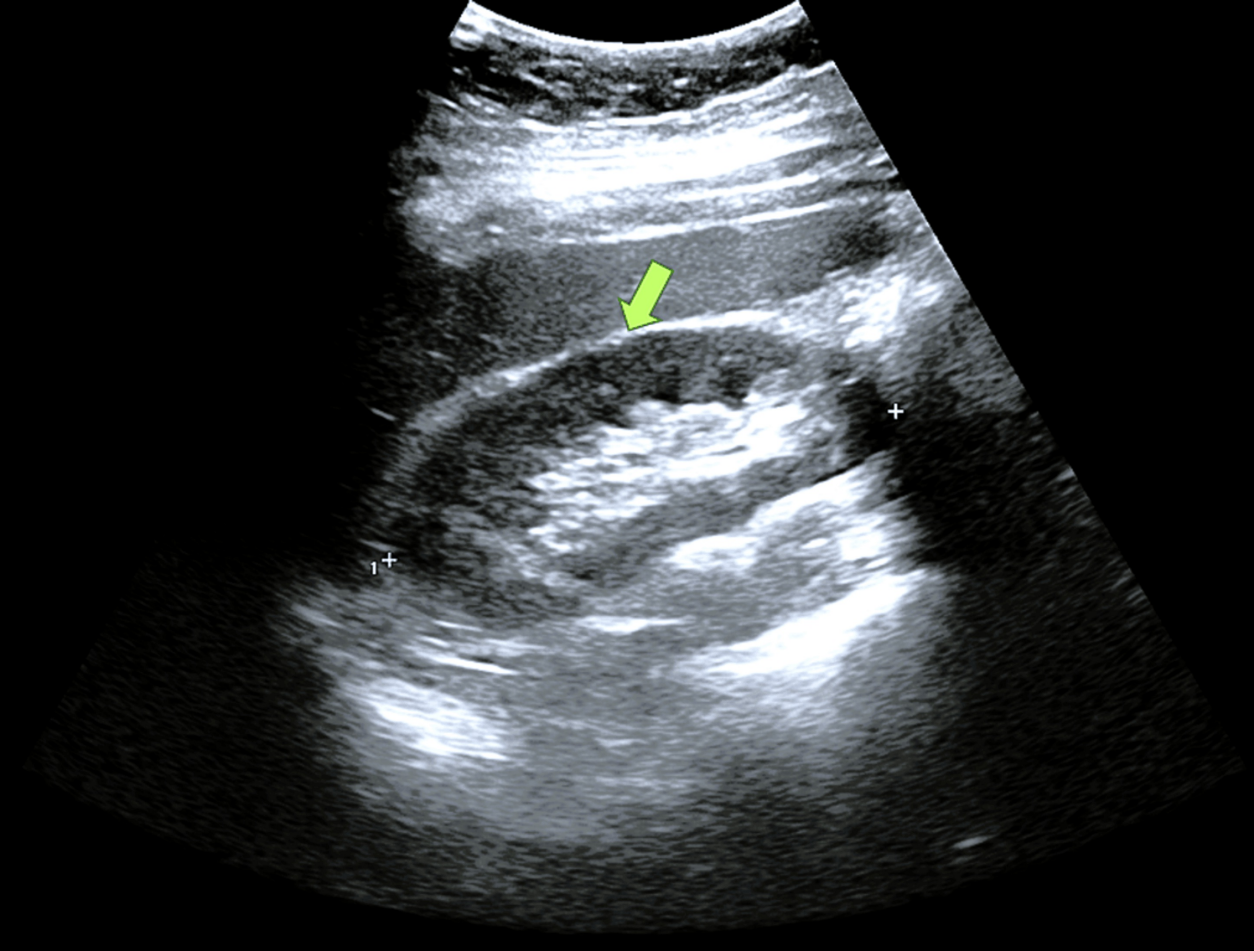
Follow-Up Ultrasound Showing Resolution of Hydroureteronephrosis Follow-up ultrasound image of the right kidney demonstrating complete resolution of previous hydroureteronephrosis (green arrow) with no residual calculus or back-pressure changes one week after spontaneous stone passage.

Repeat ultrasound one week later showed resolution of hydroureteronephrosis (Figure 4).

## Discussion

Sometimes a small stone in the lower ureter can cause pain felt mainly in the scrotum or testis because the same spinal nerves (T10-L2) supply both areas through the genitofemoral and ilioinguinal nerves [1-3]. When there is no flank tenderness or urinary complaint, the presentation can easily be mistaken for a local scrotal problem [4,5]. In such cases, if testicular ultrasound appears normal, the next step is to image the kidneys and bladder as advised in the European and American Urological Association guidelines for evaluating renal colic [6,7].

Ultrasound done in a clinic or community setup is quick, inexpensive, and free of radiation. It can show mild hydronephrosis or a small distal stone even outside a hospital [8,9]. In our case, the renal and bladder ultrasound was performed by a qualified radiologist. Identifying the cause early helps direct the right treatment. Most ureteric stones of five millimetres or less pass spontaneously within two to four weeks [10,11]. Good hydration, adequate pain relief with anti-inflammatory drugs, and an α-blocker such as tamsulosin usually help the stone move faster and reduce painful episodes [12-15]. A repeat ultrasound after some days confirms that the stone has cleared and that kidney function is recovering [16,17].

In our setting, CT imaging was not performed as it is not easily available locally and often requires travel to a tertiary centre, which increases cost and inconvenience. Many patients in small-town or semi-urban areas are not willing for expensive imaging such as CT due to financial constraints and travel burden. Ultrasound, performed by a trained radiologist, provided clear diagnostic findings and was repeated after one week to confirm stone passage and resolution of hydronephrosis. The patient’s full clinical recovery further supported diagnostic accuracy. While CT remains the gold standard for small stone detection, ultrasound can serve as a safe and practical first-line tool in resource-limited settings, provided that proper follow-up and clinical monitoring are done.

Although testicular pain from a ureteric stone has been described before, it remains uncommon in small primary-care clinics [18,19]. This case shows that simple imaging, clear communication, and watchful follow-up can allow safe conservative care without unnecessary antibiotics or referral. Recognising this pattern early saves the patient extra visits, worry, and cost while maintaining excellent recovery and satisfaction [20].

## Conclusions

This case shows how a distal ureteric stone can sometimes present only with testicular pain, without any back pain or urinary symptoms. Such cases can easily confuse doctors during the first visit. In many small centres and clinics where CT is costly or not available, an ultrasound done by a radiologist is a simple and safe first investigation. With careful follow-up and repeat ultrasound, small stones can pass naturally with good hydration, pain relief, and an alpha-blocker such as tamsulosin. Early diagnosis through ultrasound can prevent unnecessary antibiotics, hospital referrals, and surgical procedures. It also helps reduce patient anxiety and financial burden, especially in primary-care and rural settings. Greater awareness among general practitioners about such unusual presentations can improve outcomes and ensure safer, more cost-effective care. Future studies from similar resource-limited setups can help to confirm these findings and support wider use of ultrasound as a reliable first-line tool.
